# Developmental Genetics of the Perianthless Flowers and Bracts of a Paleoherb Species, *Saururus chinensis*

**DOI:** 10.1371/journal.pone.0053019

**Published:** 2013-01-30

**Authors:** Yin-He Zhao, Zachary Larson-Rabin, Guo-Ying Wang, Michael Möller, Cheng-Yun Li, Jin-Peng Zhang, Hong-Tao Li, De-Zhu Li

**Affiliations:** 1 Germplasm Bank of Wild Species, Kunming Institute of Botany, Chinese Academy of Sciences, Kunming, China; 2 College of Agronomy, and Key Laboratory for Plant Pathology of Yunnan Province, Yunnan Agricultural University, Kunming, China; 3 Institute of Crop Sciences, Chinese Academy of Agricultural Sciences, Beijing, China; 4 Royal Botanic Garden Edinburgh, Edinburgh, Scotland, United Kingdom; University of Nottingham, United Kingdom

## Abstract

*Saururus chinensis* is a core member of Saururaceae, a perianthless (lacking petals or sepals) family. Due to its basal phylogenetic position and unusual floral composition, study of this plant family is important for understanding the origin and evolution of perianthless flowers and petaloid bracts among angiosperm species. To isolate genes involved in *S. chinensis* flower development, subtracted floral cDNA libraries were constructed by using suppression subtractive hybridization (SSH) on transcripts isolated from developing inflorescences and seedling leaves. The subtracted cDNA libraries contained a total of 1,141 ESTs and were used to create cDNA microarrays to analyze transcript profiles of developing inflorescence tissues. Subsequently, qRT-PCR analyses of eight MADS-box transcription factors and *in situ* hybridizations of two B-class MADS-box transcription factors were performed to verify and extend the cDNA microarray results. Finally, putative phylogenetic relationships within the B-class MADS-box gene family were determined using the discovered *S. chinensis* B-class genes to compare K-domain sequences with B genes from other basal angiosperms. Two hundred seventy-seven of the 1,141 genes were found to be expressed differentially between *S. chinensis* inflorescence tissues and seedling leaves, 176 of which were grouped into at least one functional category, including transcription (14.75%), energy (12.59%), metabolism (9.12%), protein-related function (8.99%), and cellular transport (5.76%). qRT-PCR and *in situ* hybridization of selected MADS-box genes supported our microarray data. Phylogenetic analysis indicated that a total of six B-class MADS-box genes were isolated from *S. chinensis*. The differential regulation of *S. chinensis* B-class MADS-box transcription factors likely plays a role during the development of subtending bracts and perianthless flowers. This study contributes to our understanding of inflorescence development in *Saururus*, and represents an initial step toward understanding the formation of petaloid bracts in this species.

## Introduction

The morphological diversity of angiosperm flowers represents a breathtaking exposition of the power of evolution. Through a process known as the ABCE model of floral morphogenesis, combinations of transcription factor proteins specify organ identities in developing flowers, typically leading to the outgrowth of sepals, petals, stamens and pistils [1]–[3]. In the ABCE model, A-type transcription factors specify the respective identities of the first and second whorl organs, i.e., the sepals and petals. The B-class genes are also involved in petal identity specification in combination with A-class genes, but they combine with C-class genes in the third whorl to specify androecium development, while the gynoecium in the fourth whorl is specified by C genes, but not by A or B genes [1]–[7]. In all four whorls, E-class genes combine with A, B or C genes to specify organ identities [3], [8], [9]. In *Arabidopsis thaliana*, there are two A genes, *APETALA1* (*AP1*) and *APETALA2* (*AP2*), two B genes, *APETALA3* (*AP3*) and *PISTILLATA* (*PI*), one C gene, *AGAMOUS* (*AG*), and four E genes *SEPALLATA1 (SEP1)*, *SEPALLATA2 (SEP2)*, *SEPALLATA3 (SEP3)*, and *SEPALLATA4 (SEP4)*. With the exception of *AP2*, these floral homeotic genes belong to MADS-box gene family, named for the MADS sequence motif that encodes a domain controlling both DNA binding and homo-dimerization or hetero-dimerization with other transcription factors [4], [10], [11], [12]. The subset of plant MADS-box genes that control floral organ identities and development, the MIKC or Type II genes, also encode three other domains that are conserved to varying degrees: an Intervening domain and a Keratin-like domain that facilitate dimerization, and a C-terminal domain that is involved both in transcriptional activation and in connecting the protein to larger protein complexes [5], [13]. The 80-amino acid K domain features several (**abcdefg**)n heptad repeats, in which **a** and **d** residues are hydrophobic, forming three amphipathic α-helices (K1, K2 and K3). These α-helices form a structure that is essential for dimerization, such as in the AP3/PI heterodimer required for specification of petal and stamen identity in developing *Arabidopsis* flowers [13]–[15].

Although most floral developmental genetics research initially focused on the model eudicots *Arabidopsis thaliana* and *Antirrhinum majus*, understanding the evolution of angiosperms will require research into the developmental genetics of flowers from diverse species across the phylogenetic tree of the angiosperm clade, with special attention given to species of the basal lineages [16]. The phylogenetic position and unusual flower composition of the basal species *Saururus chinensis* render it attractive for the study of flower developmental genetics in general and MADS-box genes in particular. This species is a member of Saururaceae, a family which is a member of an early divergent angiosperm lineage (magnoliids) [16]–[18]. Inflorescences emerge sequentially, each comprising a single spike with 80–150 flowers that mature in acropetal succession with bracts subtending each individual flower. The flowers are white with six stamens in three pairs, four carpels, and no perianth organs [19]. Unlike many other perianthless species in which the first and second whorls are entirely absent, the subtending bract primordium arises roughly concurrently to floral meristem inception in *S. chinensis* [19]. Petaloid bracts are found in other species, such as *Justicia brandegeeana*; these bracts likely perform the pollinator-attraction role of true petals, and their petaloidy probably results from ectopic expression of petal development genes [20]. The “sliding boundary” model proposes that petaloid features in sepals and bracts result from enlargement of the expression domain of B-class genes [20], [21], [22]. Indeed, ectopic expression of *AP3* and *PI,* with the addition of *SEP3* expression, was sufficient to give *Arabidopsis* leaves petaloid characteristics [5]. However, natural development of petaloid tissue does not always require expression of *PI* and *AP3* homologues [23], and petals have arisen independently several times during angiosperm evolution [22]. In *S. chinensis,* understanding the loss of perianth tissue and evolution of showy bracts could shed light on the derived origin of such petaloid, but non-petal, structures.

The origin of petals has been a popular topic of research and theory, and the function and evolution of the B-class genes have been central to such research because of their roles in petal development [22], [24]. The history of B gene functional evolution is complex, however, since B gene expression has also been found in gymnosperm androecia and in other non-petal angiosperm organs, such as the staminodes of Aquilegia sp. [25]–[28]. The perianthless paleoherb *S. chinensis* provides a unique opportunity to study the genetics of floral development in a basal angiosperm, whose bracts are at least analogs of petals and perhaps the petaloidy of the bracts that subtend the flowers could share functional homology with second whorl petals of other taxa. In this work, we constructed tissue-specific cDNA libraries using suppression subtracted hybridization (SSH), and assigned the *S. chinensis* genes to functional categories. We then compared expression profiles of bracts subtending flowers, stamens and carpels using cDNA microarrays, and validated the results for key genes by qRT-PCR. We also performed phylogenetic analysis to compare *S. chinensis* B-class genes with their homologues in other plant species. Finally, we investigated B-class gene expression and transcript localization within subtending bracts and floral organs by *in situ* hybridization.

## Results

### Construction of forward and reverse subtracted cDNA libraries

To isolate transcripts with differential abundance between *S. chinensis* leaves, subtending bracts, and floral organs, an inflorescence-enriched cDNA library was constructed using suppression subtractive hybridization (SSH). Following two rounds of hybridization and amplification, a set of forward and reverse subtracted cDNAs of high quality were cloned to construct the forward and reverse SSH libraries. The positive control template in the Advantage cDNA PCR Kit was used to ensure the efficiency of the PCR system. In addition, subtraction efficiency was verified by analysis of levels of the conserved *α–tubulin* control. The *α-TUBULIN* (JK704891) band could be observed after 18 cycles of amplification from the unsubtracted cDNA population, while 28 cycles were necessary to amplify an observable *α-TUBULIN* product from the subtracted cDNA population.

### Sequencing, annotation, and assignment of putative functions to ESTs

**Figure 1:**
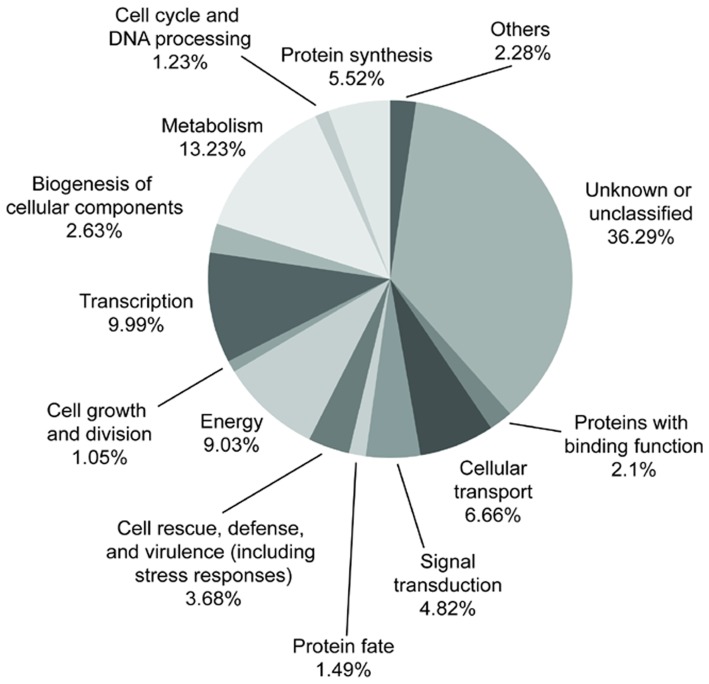
Functional classification of unique *S. chinensis* ESTs according to the MIPS Functional Catalogue scheme.

We sequenced a total of 1,083 clones from the forward library and 500 clones from the reverse library, obtaining 935 and 458 ESTs longer than 100 bp, respectively. Their lengths ranged between 100 to 800 bp, but were predominately between 300 and 600 bp. ESTs showing at least 95% identity over 40 or more bases were classed into the same cluster using SEQUENCHER (www.genecodes.com). The 935 forward ESTs were assembled to make 82 contigs and 684 singletons representing up to 766 different genes. The 458 reverse ESTs formed 42 contigs and 333 singletons representing up to 375 different genes. The percentages of unique sequences in the forward and reverse libraries were 89.29% and 88.8%, respectively. The 1,141 total ESTs were classified into 14 MIPS functional categories (http://mips.helmholtz-muenchen.de/plant/athal/) (Figure 1). Within these groups, for 414 ESTs (36.29% of the total) no match was found among Genbank accessions, because they were too divergent from published sequences. Of the genes with recognizable similarity to known genes, 114 were assigned putative function in transcription (9.99%), 151 in metabolism (13.23%), 103 in energy (9.03%), 42 in cell rescue, defense and virulence (3.68%), 76 in cellular transport (6.66%), 63 in protein synthesis (5.52%), 24 in the binding of proteins or cofactors for structural or catalytic purposes (2.1%), 55 in signal transduction (4.82%), 30 in cellular component biogenesis (2.63%), 12 in cell growth and division (1.05%), 14 in cell cycle progression and DNA processing (1.23%), and 17 in protein fate including folding, modification, and localization (1.49%). Sequences from another 26 genes showed putative functional assignments outside these categories (2.28%).

### Genes showing differential transcript abundance between developing inflorescence organs and leaves

To compare the differences in transcript abundance between developing leaves and inflorescence tissues, a custom cDNA microarray was designed and hybridised with tissue-specific transcripts. Total RNA was isolated from subtending bracts, carpels and stamens at various stages of inflorescence development before anthesis young seedling leaves, as well as from developing upper seedling leaves. Comparing the inflorescence tissue transcript profiles with the transcripts of the leaves, we identified 277 differentially expressed genes, among which 33% (47) were up-regulated and 67% (96) were down-regulated in subtending bracts, 60% (113) were up-regulated and 40% (76) were down-regulated in stamens, and 58% (109) were up-regulated and 42% (80) were down-regulated in carpels (Table S2).

To assign putative functions to the differentially expressed genes, the transcript sequences were grouped into 10 MIPS functional categories according to the Functional Catalogue (http://mips.gsf.de). Among the 277 differentially expressed genes, 176 could be grouped into at least one functional category, while the remaining 101 genes showed no significant hits to any sequences published in GenBank. The most prevalent functional categories, and the numbers and proportion of genes assigned to them, were as follows: transcription (14.75%), energy (12.59%), metabolism (9.12%), protein-related function (8.99%), and cellular transport (5.76%) (Table 1). We found significant transcript abundance differences among the inflorescence organs. Of the genes expressed differentially in subtending bracts, the most abundant functional categories included energy (16.78%), metabolism (11.12%), transcription (9.79%), protein-related function (9.09%), and cellular transport (7.69%) (Table 1). These categories were also the most abundant in stamens, but at different proportions, with most genes assigned to energy (13.22%), followed by transcription (12.7%), metabolism (11.11%), protein-related function (8.99%), and cellular transport (4.23%) (Table 1). Conversely, in carpels the most abundant functional category was transcription (12.7%), followed closely by energy (12.17%), metabolism (11.11%), protein-related function (8.99%), and cellular transport (5.82%) (Table 1). As expected, many genes related to photosynthesis, including chlorophyll and other chloroplast components, were down-regulated in the inflorescence organs compared to young leaves (Table S2).

**Table 1:** MIPS Functional Category assignments of *S. chinensis* genes differentially expressed during inflorescence tissue development relative to seedling leaf development.

MIPS Functional Category	Total		Subtending Bracts		Stamens		Carpels	
Transcription	41	14.75%	14	9.79%	24	12.70%	24	12.70%
Energy	35	12.59%	24	16.78%	25	13.22%	23	12.17%
Metabolism	27	9.12%	16	11.12%	21	11.11%	21	11.11%
Cellular transport	16	5.76%	11	7.69%	8	4.23%	11	5.82%
Biogenesis of cellular components	9	3.24%	2	1.40%	7	3.70%	6	3.17%
Signal transduction	9	3.24%	5	3.50%	5	2.65%	6	3.17%
Cell rescue, defense, and virulence	11	3.96%	5	3.50%	7	3.71%	6	3.17%
Protein-related function (protein synthesis, binding, and fate)	25	8.99%	13	9.09%	17	8.99%	17	8.99%
Cell cycle, cell growth, andDNA processing	3	1.08%	2	1.40%	4	2.12%	3	1.59%

### Significantly up-regulated transcription factor homologues

Relative to the transcriptome of developing seedling leaves, transcripts of a host of transcription factor genes were found to show differential abundance among the transcriptomes of developing carpels, stamens, and subtending bracts (Table 2). The significantly up-regulated transcription factor genes included *ScAP1*, *ScSEP1*, *ScAG, ScAGL6, ScPI* and *ScAP3*, in addition to genes generically identified as MADS-box transcription factors, and members of the zinc finger, basic helix-loop-helix (bHLH*)*, and DnaJ protein families (Table 2). The relative transcript abundances of these genes, however, differed between tissue types. For example, *ScSEP1* relative transcript abundance was 10.85 in stamens but only about 4.9 in carpels and subtending bracts. Stamens also showed especially high transcript abundance of an unknown bHLH transcription factor, two zinc-finger transcription factors and three *MYB* transcription factors, two of which were designated as *ABORTED MICROSPORES (AMS)* homologues. In developing carpels, one of the *AMS-like* genes was much more abundant than in the stamen samples (Accession no. JK705102), and a zinc finger protein similar to a putative oncogene in humans (*ZNF652*) showed very strong up-regulation in carpels.

**Table 2:** Subset of transcription factors determined to be up- or down-regulated in inflorescence tissues, relative to their transcript abundance levels in developing seedling leaves.

Accession No	Putative Identification	Relative Transcript Abundance		
Up-regulated genes		Subtending Bracts	Stamens	Carpels
JK705099	NAC transcription factor	1.47	1.11	2.61
JK705137	*AGL6-like*	1.13	0.07	2.92
EG530712	*AP1-like*	3.19	1.41	2.30
JK705246	MYB transcription factor	0.93	6.45	0.28
JK705297	*AP1-like*	3.61	0.03	1.9
JK704641	bHLH transcription factor	2.16	19.19	0.67
EG530714	*SEP1-like*	4.91	10.85	4.93
EG530716	*AGL6-like*	4.46	0.49	3.57
EG530715	putative MADS-box protein	0.84	3.27	0.28
EG530710	*ScPI-A*	1.11	2.08	0.91
EG530711	*ScPI-B*	0.81	2.01	1.70
EG530709	*ScAP3-A*	2.36	3.26	1.11
JK704951	homeobox transcription factor	0.09	3.31	0.26
EH662329	*ScAP3-B*	1.21	8.65	4.91
JK705019	*AGAMOUS-like*	1.41	8.71	2.61
JK705102	*AMS (ABORTED MICROSPORES)*	0.81	3.42	35.76
JK704608	*AMS (ABORTED MICROSPORES)*	1.25	8.82	1.36
JK705161	putative zinc finger protein	1.43	0.09	8.67
JK705224	similar to zinc finger protein 23	0.43	3.20	0.32
JK704646	DNA binding/transcription factor	1.34	0.19	2.39
JK704816	similar to zinc finger protein 652	1.16	42.90	66.09
JK704903	DnaJ protein	2.00	1.28	1.31
JK704904	zinc finger (CCCH-type) protein-l	1.53	10.28	2.23

Down-regulated genes	Putative Identification	Subtending Bracts	Stamens	Carpels
JK704691	*AUXIN RESPONSE FACTOR 3*	1.44	0.97	0.43
JK704682	Similar to *AUXIN RESPONSE FACTOR 9*	0.38	0.32	1.85
JK704881	similar to zinc finger protein	1.10	0.02	1.11

There were also some genes with transcript abundance levels nearly equal between subtending bracts, stamens, and carpels; these included a putative NAC transcription factor. Examples of genes down-regulated in inflorescence tissues relative to seedling leaves included homologues to auxin response factors and several zinc finger proteins (Table 2). Surprisingly few transcripts belonging to the microspore-specific transcriptome were discovered in our analysis. This may be because microsporogenesis occurred largely post-anthesis in these plants, or perhaps the *S. chinensis* genes involved in microsporogenesis show little sequence similarity to known microspore development genes.

### Support of microarray results by real-time quantitative PCR

**Figure 2:**
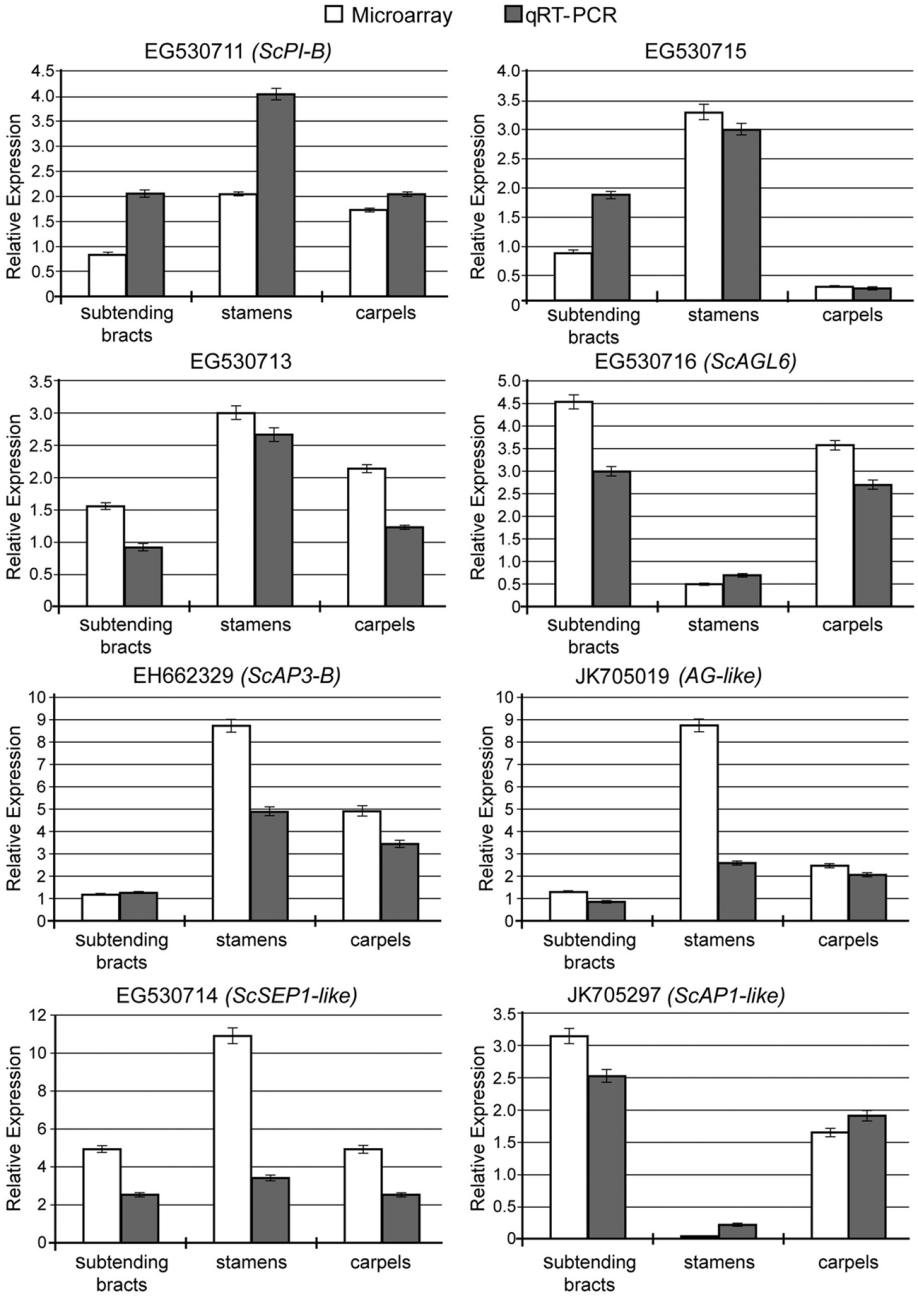
Validation of microarray results by qRT-PCR for selected genes (EG530711 *(ScPI-B)*, EH662329 *(ScAP3-B)*, EG530714 *(ScSEP1-like)*, EG530715, EG530716 *(ScAGL6)*, D945 *(ScAG-like)*, D1420 *(ScAP1-like)* and EG530713). *S. chinensis α-TUBULIN* (JK704891) was used as an internal control for normalization of the template cDNA. Each independent experiment was performed three reactions, and the error bars represent the standard deviation of three independent experiments. In each graph, the white bars represent the relative expression found in the microarray experiments, and the grey bars represent the relative expression determined by normalised qRT-PCR.

To evaluate the accuracy and reproducibility of the microarray results, eight MADS-box transcription factors (EG530711, EG530713, EH662329, EG530714, EG530715, G530716, G530719 and JK705297) were selected for confirmation by qRT-PCR (Figure 2). The qRT-PCR results showed transcript abundance trends for these genes that were largely consistent with the microarray results. There were some discrepancies, however, such as in the case of the EG530711 (*ScPI-B*) gene, which showed marked up-regulation only in stamens in the microarray analysis but seemed to be up-regulated in all inflorescence organs by qRT-PCR (Figure 2), when considering an equal or above 2-fold up-regulation as significance threshold. This difference is perhaps due to a higher sensitivity of qRT-PCR compared to the microarray.

### Phylogenetic analysis

**Figure 3:**
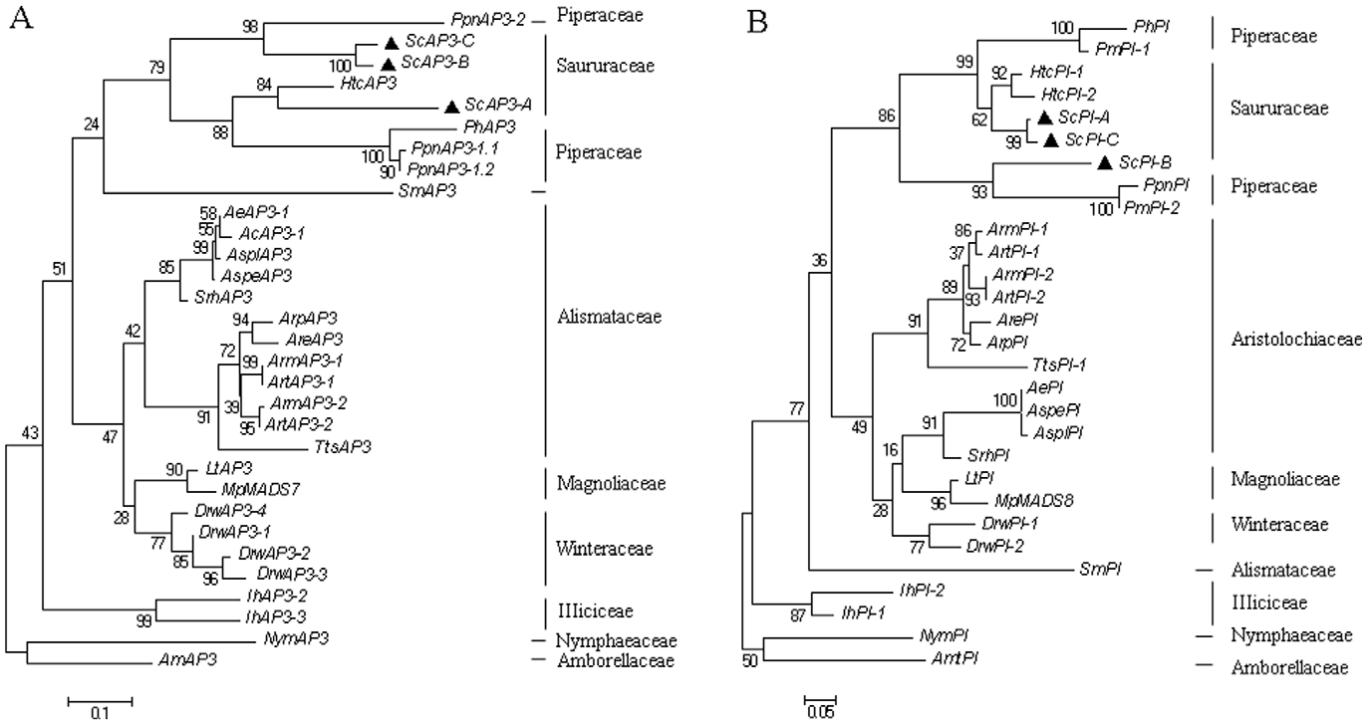
Maximum likelihood tree of B-class putative MADS-box transcription factors from *S. chinensis* and other basal angiosperm species. (A) Phylogenetic reconstruction of *AP3* homologs from basal angiosperms using 226 sites. (B) Phylogenetic reconstruction of *PI* homologs from basal angiosperms using 420 sites. Numbers along branches represent bootstrap support values.

The maximum likelihood analysis of the B-class gene nucleic acid alignments supported the identification of two *S. chinensis* ESTs as *APETALA3*-family homologues, with EG530709 as *ScAP3-A* and EH662329 as *ScAP3-B*. A third, previously isolated *AP3* homologue, *MADS651* (GenBank accession AY057378, D.Z.L. unpublished data; [29]) grouped with these two clones and thus was designated *ScAP3-C* (Figure 3A). *ScAP3-C* and *ScAP3-B* were strongly supported (100% bootstrap, BS) as sister to *PpnAP3-2* from *Piper nigrum*. *ScAP3-A* was placed close to a *Houttuynia cordata AP3* homologue with moderate bootstrap support (84% BS); it also fell closely to two *AP3-1* homologues from *P. nigrum* and the *AP3* homologues from *Peperomia hirta* (88% BS) (Figure 3A).

The gene homology for *S. chinensis PISTILLATA* genes was also supported, with EG530710 representing *ScPI-A*, EG530711 *ScPI-B*, and *MADS658 ScPI-C* (GenBank accession AJ419691, D.Z.L. unpublished data; [29]) (Figure 3B). The putative *PI* homologues from *S. chinensis* fell in one clade with the *PI* homologues from *H. cordata*, *P. hirta*, *Piper magnificum*, *P. nigrum* with moderate bootstrap support (86% BS). Within this clade, *ScPI-B* was sister to *PmPI-2* and *PpnPI* (93% BS). *ScPI-C* and *ScPI-A* were also paired with high bootstrap support (99% BS), and were placed closer to the putative *PI* genes from *H. cordata* than to *ScPI-B* (Figure 3B).

### *In situ* hybridization analysis of two B-class MADS-box transcription factors in *S. chinensis* inflorescences

**Figure 4:**
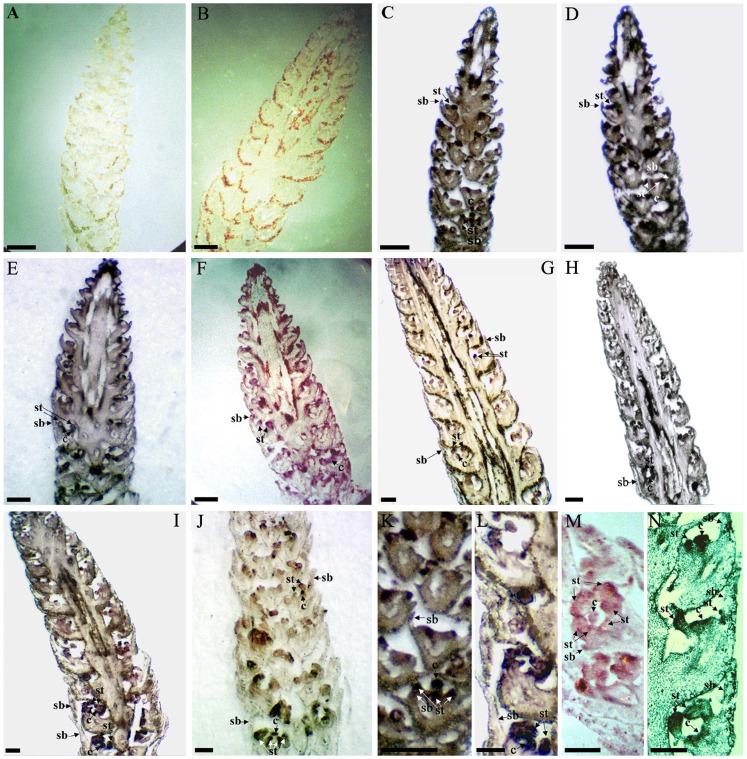
*In-situ* hybridization analysis of EH662329 (*ScAP3-B*) and EG530711 (*ScPI-B*) in longitudinal sections of developing *S. chinensis* inflorescences. (A and B) *In-situ* hybridization with EH662329 and EG530711sense probe as negative controls, respectively. (C, E, G, I, K and L) *In-situ* hybridization using EH662329 anti-sense probe. (D, F, H, J, M, N) *In-situ* hybridization using EG530711 anti-sense probe. (C, D, K), (E, F, M), (G, H, N), and (I, J, L) represent four progressive stages in inflorescence development. sb, subtending bracts; st, stamens; c, carpels. Scale bars  = 500 µm.

To examine the spatial expression patterns of the putative MADS-box transcription factors isolated from *S. chinensis*, *ScAP3-B* (EH662329) and *ScPI-B* (EG530711), we performed RNA *in situ* hybridization on developing inflorescence tissues. Sense-strand control probes of *ScAP3-B* and *ScPI-B*, derived from ESTs EH662329 and EG530711, respectively, showed no signal background in any of the tissues (Figure 4A and B). *ScAP3-B* antisense probes were localized in the primordia of subtending bracts, stamens and carpels (Figure 4C, E, and K) in the early stages of floral development. Expression in the subtending bracts appeared somewhat localized in the bract tips (Figure 4C, and K), but was later restricted to the nascent stamens and carpels, showing stronger signals during later stages of floral development (Figure 4G, I and L). Probes specific for *ScPI-B* (EG530711) were also detected in primordia of all three inflorescence organs in the early stages of floral development (Figure 4D, F, M), and high expression levels were again restricted to stamens and carpels shortly thereafter (Figure 4H, J and N).

## Discussion

This study represents the first transcriptomic analyses of *S. chinensis,* a paleoherb species whose perianthless flowers, showy bracts, and phylogenetic position were expected to yield fresh insights to our understanding of angiosperm evolution. Given that genomic information was lacking in the Saururaceae, the EST library construction and expression analyses described here provide an important contribution to the floral developmental genetics of paleoherb species. Despite this species' phylogenetic distance from model angiosperms, most of the *S. chinensis* genes described here show sequence similarity and, especially for *ScAP3* and *ScPI* genes (Figure 1, Table 1). In addition, more than a third of the genes discovered in this study represent novel genes or, at least, hypothetical genes of unknown function. Besides those classed as hypothetical and unknown genes or metabolism-related genes, the largest group was the transcription-related genes (Figure 1, Table 1).

Because many floral development genes are expressed at low levels, suppression subtractive hybridization (SSH) has proven to be useful for their isolation [30]. The analysis of our SSH results indicated that our procedure was successful in isolating transcripts involved in *S. chinensis* flower development. More than 98% of sequenced genes were represented by just 1–3 ESTs, indicating that the cDNA library had been efficiently normalised and the transcript abundance of highly expressed genes had been dramatically reduced, while low-abundance transcripts had been comparatively enriched. This success facilitated the isolation of a total of 1,141 ESTs, and led to the subsequent identification of 277 differentially expressed genes using the custom cDNA microarray. Although these ESTs likely represent only a fraction of the total transcriptome of the developing inflorescence, they will nonetheless provide a useful framework for future investigations of paleoherb plants. The cDNA microarray analysis enabled pairwise comparisons of the transcript profile of developing vegetative leaves with the transcriptomes of the subtending bracts, stamens and carpels. Relative to developing leaves, many transcription factors were differentially up-regulated in the subtending bracts and floral organs (Table 2 and Table S2). A similar trend was observed in rice panicles and *Arabidopsis* inflorescences, in which 30.4% and 21.2% of early-expressed transcripts were identified as putative transcription factors, respectively [31], [32]. Sequence comparisons showed that at least 10 of these early-expressed transcription-related genes encoded MADS-box transcription factors, the critical regulators of flower development [3], [8] (Table 2; Table S2). In this study, anther tissues were retained to maximise the comprehensiveness of the stamen transcriptome, yet surprisingly few recognizable microspore transcripome genes were discovered. This suggests that microsporogenesis may occur later than the range of maturity collected in this study (i.e., microsporogenesis occurs at or after anthesis). Another possibility is that the transcripts comprising the microspore transcriptome bear little sequence similarity to known microsporogenesis genes.

*AP3* and *PI* homologues determine petal and stamen identity in many angiosperm species, and thus these genes are central to the evolution of the angiosperm petal [3]. Often restricted to petals and stamens in the core eudicots, B-class genes have also been shown to be expressed in male reproductive organs in gymnosperms such as *Gnetum*, plants that do not produce petals; this suggests that ancestral B genes were male-function specific and that petal functions arose during angiosperm evolution through neofunctionalization of B-gene duplicates [25], [26]. In basal eudicots like those in Ranunculaceae, B genes were found to be extensively duplicated, with the function of some paralogues separated from perianth development [33], [34]. In several basal angiosperm species, B-class genes have been shown to be expressed in a larger domain that includes the first whorl, a phenomenon likely responsible for petaloid tepals instead of differentiated sepals and petals [28], [35].

Bracts are leafy organs that subtend flowers or flower clusters on the inflorescences of many plant species. They often contain chloroplasts and can contribute to the photosynthetic capacity of the plant [36], although bract development has been convergently suppressed in diverse clades [37]. The bracts of some species are colourful and showy, functioning to supplement or substitute perianths (e.g., *Bougainvillea* and the poinsettia). In fact, petals can be considered to represent the developmental continuation of bracts in some higher eudicots [24], [38]. It has been suggested that gene networks controlling colour and other petaloid characteristics in petals or tepals are ectopically expressed in such showy bracts [22]. However, although presence of petals or petaloid perianth tissue generally corresponds with expression of B-class genes, whether in monocots or dicots [39], it is not a strict requirement and natural petaloid tissue has been observed in the absence of B-class gene expression [23]. Regarding the white bracts subtending flowers of *S. chinensis,* we hypothesized that expression of B-class genes may be responsible for the petaloid character. We compared the set of transcripts expressed in the white subtending bracts of *S. chinensis* with those expressed in seedling leaves and in floral organs. Indeed, we discovered that *ScAP1, ScAP3-A, ScAP3-B*, *ScPI-A* and *ScPI-B* transcription factors were up-regulated in developing *S. chinensis* subtending bracts (Figure 2,and 4, Table 2), while genes involved in photosynthesis were down-regulated (Table S2). This suggests that the developmental genetic program of *S. chinensis* bracts has evolved away from foliar photosynthetic functions and toward floral display function. A MADS-box gene with similarity to *AGAMOUS-LIKE6 (AGL6,* EG530716*)* was also expressed in *S. chinensis* subtending bracts and carpels; strongly activated *AGL6* has been shown to induce bract production in the normally ebracteate *Arabidopsis* [40]. Another homolog of *AGL6* (JK705137), also showed expression in carpels (Table 2 and Table S2), suggesting some functional conservation of those genes in *S. chinensis*.

This study revealed the *S. chinensis* B-class genes to include at least three paralogues of *AP3* and three paralogues of *PI*, suggesting two gene duplication events for each gene lineage in this species (Figure 3). The observed sequence divergence possibly resulted from relaxed selection following gene duplication, since such a process has been indicated for Piperales, including Saururaceae [29]. Indeed, the B-class genes were found to be expressed in all three inflorescence tissue types, but varied in transcript abundance levels. The strong expression of *ScAP3-B*, and to a lesser degree *ScPI-B*, in carpels (Table 2, Figure 2) may be consistent with a hypothesis of neofunctionalization, and may suggest gain of function of these paralogues in carpel development [34]. *ScAP3-A* and *ScPI-A* mainly expressed in stamens (Table 2), it suggested that *AP3* and *PI* lineages maybe experience gene duplication and subfunctionalization.

To further explore the timing and pattern of expression of these of B-class paralogues, *in situ* hybridization was performed using *ScAP3-B* and *ScPI-B* as probe templates. During early stages of floral development, signals for both of the *ScAP3-B* and *ScPI-B* were detected in the primordia of subtending bracts, stamens and carpels (Figure 4C, D, E, F, K and M), but in the later stages, *ScAP3-B* signals remained largely restricted to the stamens and carpels (Figure 4G, I and L). The signal of *ScPI-B* was especially strong in the stamens, but was detected in the carpel primordia as well (Figure 4H, J and N). These findings were consistent with previous observations of an expanded expression pattern for B-class genes in the basal angiosperms [28], [35], [41], [42]. *ScAP3-C* and *ScAP3-B* showed 2-aa deletions in the c and d positions of the 3′end of the K1 α-helix (Figure S1), also corresponding to the 3′ end of the third exon [29], [43], [44]. One possible effect of the deletion is that it may have shifted alternative splice sites [29], [43]. Considering what is known about their interactions in *Arabidopsis*, this may have considerably altered dimerization capabilities [29], [44].

Studies of *PI* homologues in basal angiosperms have revealed a characteristic 12-nucleotide deletion was hypothesized to be a synapomorphy of the rest of angiosperms following the divergence of the ANITA grade; the deletion event apparently occurred after the split of the Nymphaeales but before the separation of the Austrobaileyales [45]. In *S. chinensis,* all three *PI* homologues (*ScPI-A, ScPI-B* and *ScPI-C*) also displayed this 12-nucleotide deletion, which was located at the beginning of the third α-helix of the K domain (Figure S2) [13], [44], [46], [47]. The deletion shifts the pattern of hydrophobic heptad repeats in this domain, probably altering protein interactions [13], [29]. Therefore, in the perianthless inflorescence of *S. chinensis*, it seems likely that AP3/PI dimerization capabilities have been attenuated by the deletion, and it seems reasonable that such protein alterations would have functional importance.

In many perianthless species, first and second whorl primordia initiate but then arrest; in Saururaceae and Piperaceae, conversely, petal and sepal primordia are completely absent throughout floral morphogenesis [48]. As such, it has been predicted that selection pressures on *AP3* and *PI* would be reduced in the perianthless Piperales, as these genes have been released from their perianth identity function [29], and the same should be true for Saururaceae. We therefore suggest that two factors, the reduced dimerization capabilities of the various *S. chinensis* AP3/PI combinations and the complete lack of perianth tissues, combined to render *S. chinensis* B-class genes especially free from evolutionary pressures. It will be interesting to determine what functions the broad expression patterns of the *AP3* and *PI* genes have maintained in this perianthless species.

In conclusion, this research has initiated the study of the developmental genetics of *S. chinensis,* providing sequence and expression data for the B-class MADS-box transcription factors and other inflorescence-related genes of this perianthless species. We found differential regulation of *S. chinensis* B-class MADS-box transcription factors that likely play roles during the development of bracts subtending flowers and perianthless flowers. Some paralogues showed an extended expression in early and late stages of carpel development and early stages of inflorescence development of bracts subtending flowers, suggesting some transfer of function. Additional studies will help to elucidate further the functions and evolutionary histories of B-class genes in the basal angiosperms.

## Materials and Methods

### Tissue collection and RNA extraction and purification

An inbreeding population of *Saururus chinensis* was cultivated in the Botanical Garden of the Kunming Institute of Botany, Chinese Academy of Sciences, Kunming, Yunnan Province, China. Subtending bracts, stamens and carpels were pooled from various floral developmental stages until anthesis. Collected tissues were immediately frozen in liquid nitrogen and stored at −80°C. Between 7 to 14 days following the initiation of inflorescence development, when the first of an inflorescence's flowers had reached anthesis, 500 flowers from 5 inflorescences were collected and their organs were removed. Because the flowers on each inflorescence matured acropetally, harvesting all of the floral organs on target inflorescences at once provided a nearly continuous series of organ maturity from 0.5 mm to nearly full size (just prior to anthesis). Five hundred developing bracts were collected at lengths of from 0.5 mm to just before anthesis (up to 3 mm). All organs of a given organ type were pooled and immediately frozen in liquid nitrogen. Due to the high number of sample organs and their diversity of developmental progression, samples were not corrected for volume; however, by collecting all flowers of each inflorescence just as the first flowers reached anthesis, there were relatively few of the most mature flowers. This may alleviate the effects of the relative over-representation of transcripts from the larger, more mature organs relative to the more abundant but less mature organs. Young upper leaves were also collected at the seedling stage and frozen. Total RNA was isolated from the tissues using TRIZOL reagent (Shanghai Huashun Co. Ltd., Shanghai, China) according to the manufacturer's protocol. The ratio of 28S:18S RNA was about 2∶1, suggesting a high total RNA quality. To remove any possible DNA contamination, the samples were treated with RNase-free DNase I (Promega). Poly-A+ RNA was purified from the isolated total RNA using an Oligotex mRNA Purification Kit (Qiagen, Valencia, CA, USA) according to the manufacturer's protocol.

### cDNA library construction

The PCR-Select cDNA Subtraction Kit (Clontech, Palo Alto, CA, USA) was used to generate a subtractive floral cDNA library using the methods of Zhao et al. [49]. The procedure was performed twice. The forward subtraction procedure used 2 mg of pooled mRNA from inflorescences at various stages of development to make the “tester” cDNA population, and 2 mg of pooled mRNA from developing seedling leaves was used to make the “driver” cDNAs. For the reverse subtraction procedure, the tester and driver mRNA sources were reversed. cDNA subtraction was performed according to the manufacturer's instructions. The secondary PCR products from both procedures were inserted directly into pGEM-T Easy vectors (Promega, Madison, WI, USA) and transferred into *Escherichia coli* DH5α cells by electroporation (Gene Pulser; Bio-Rad, Hercules, CA, USA). Transformed *E. coli* were selected on LB/ampicillin/IPTG/X-Gal plates and white colonies were cultured overnight at 37°C in 96-well plates with 400 μl LB medium plus ampicillin per well. The cDNA inserts were amplified by polymerase chain reaction (PCR) (Perkin-Elmer GeneAmp PCR System 9600, Waltham, MA, USA) using primers flanking the cDNA insertions (Forward primer: 5′-TCGAGCGGCCGCCCGGGCAGGT-3′; Reverse primer: 5′-AGCGTGGTCGCGGCCGAGGT-3′). Thermo-cycling conditions included an initial denaturation at 94°C for 3 min, followed by 28 cycles of 95°C for 10 s, 68°C for 3 min, and a final elongation step at 72°C for 7 min.

### Transcript sequencing and annotation

The *S. chinensis* cDNA library of 1,583 randomly collected clones was sequenced by Hua Da Genomic Company (Beijing, China). After vector and adaptor sequences were trimmed, the sequences were assembled using SEQUENCHER (www.genecodes.com), with minimum overlaps of 40 bases possessing at least 95% identity [50]. From the 1,528 clones, 1,141 ESTs were produced. To determine the function of these ESTs, nucleic acid and protein homology BLAST searches were performed against the NCBI database (http://www.ncbi.nlm.nih.gov/database). Sequence matches with e-values smaller than 1×10^−5^ across more than 100 nucleotides were considered significant [50] and homologous sequences were collected for further analyses. Functional classification of the ESTs was carried out according to the MIPS FunCat annotation scheme used for *Arabidopsis* (http://mips.gsf.de/proj/thal/db/index.html).

### cDNA microarray construction and analysis

Forward and reverse subtracted cDNA libraries were used to design a custom microarray (Hua Da Genomic Company, Beijing, China). Microarray probes were derived from the 1,141 ESTs according to Zhang et al. [51] (2007) with minor modifications. The probes were generated by reverse transcription of 50 µg of total RNA using the SuperScript Indirect cDNA Labeling System (Invitrogen, Carlsbad, CA, USA). The reverse transcription reaction also contained 1 ng of Lambda poly-A+ RNA-A (TX802; Takara, Kyoto, Japan) as an external control. Lambda DNA (TX803; Takara, Kyoto, Japan) was used as a control template, and SARS virus genes and distilled water were used as negative controls. Probes derived from RNA of seedling leaves were labeled with Cy3 fluorescent dye, and probes of bracts subtending flowers and floral tissue transcripts were labeled with Cy5 fluorescent dye. Equivalent quantities of Cy3- and Cy5-labeled probes were pooled, purified, dried under vacuum according to Zhang et al.[51] (2007), and resuspended in 1× hybridization solution (5× standard saline citrate, 0.1% SDS, 25% formamide, and 0.1 mg/mL of denatured salmon sperm DNA). A common reference design was adopted in our cDNA microarray experiment [52], and two biological replicates for each time point were completed. Pre-hybridization, hybridization, and post-hybridization washes were carried out in accordance with the UltraGAPS Coated Slides instruction manual (Corning, Lowell, MA, USA). After being washed, the slides were scanned using an Axon GenePix 4100B scanner (Molecular Devices, Union City, CA, USA) at a resolution of 10 μm. Laser and photomultiplier tube voltages were adjusted manually to minimize background, to reduce the number of spots that showed saturated signal values, and to bring the signal ratio of the majority of control genes as close to 1.0 as possible. Spot intensities were quantified using Axon GenePix pro 6.0 software (Molecular Devices, Sunnyvale, CA, USA). The intensity of each channel for individual spots was calculated by determining the median value, and background fluorescence was calculated on the basis of the fluorescence signal of the negative control genes. The Lambda control template DNA fragment was used to normalize the fluorescence intensities of the two labeling dyes for dye bias reduction. The spots with a ratio ≥2 were judged significantly up-regulated and analyzed with Hierarchical Clustering Explorer 3.0 (http://www.cs.umd.edu/hcil/hce/hce3.html). Only those genes with ≥2-fold or ≤0.5-fold differences in expression were considered to be differentially expressed.

### Real-time quantitative PCR analysis

Total RNA samples of the seedling leaves, stamens, carpels and bracts subtending flowers were pooled from inflorescences at anthesis, and subjected to DNAse treatments as above to remove genomic DNA contamination before reverse transcription, and then reverse transcribed with oligo-dT and reverse transcriptase (Promega, Madison, WI, USA) following the supplier's protocols. To validate the cDNA microarray results for eight putative MADS-box transcription factors, real-time quantitative PCR (qRT-PCR) for the examination of the expression of the eight putative MADS-box transcription factors was performed on an ABI Prism 7500 HT sequence detection system (Applied Biosystems, Carlsbad, CA, USA) with three biological and three technical replicates. Primers were designed using Primer5 and synthesised by ShengGong Co. (Shanghai, China), and are listed in Table S1. An *α-TUBULIN* gene (JK704891) was used as internal control for normalization of the template cDNA. qRT-PCR reactions used 1× SYBR Green I PCR Master Mix (Applied Biosystems, Carlsbad, CA, USA) containing 200 nM of each primer and 1μl 1∶10 diluted total cDNA. The PCRs were performed using the following program: 1 cycle at 50°C for 2 min, 1 cycle at 95°C for 10 min, and then 40 cycles of 95°C for 30 s, 60°C for 30 s, and 72°C for 18 s. Following amplification, melting curve analyses were performed at 95°C for 10 min followed by 40 cycles of 95°C for 30 s, 60°C for 30 s, and 72°C for 20 s. The data collected during each extension phase were analysed using SDS2.1 (Applied Biosystems, Carlsbad, CA, USA). The transcript abundance of the eight putative MADS-box transcription factors was calculated using the relative 2^−△△*C*T^ analytical method [53]. Each sample was done in triplicate, and the mean and standard deviation of the three independent experiments were calculated.

### Phylogenetic analysis

The unique ESTs reported in this paper were deposited in the NCBI EST GenBank database (http://www.ncbi.nlm.nih.gov/dbEST) with the following accession numbers: EH662329 − EH662331, EG530707 − EG530716, JK704596 − JK704704, and JK704711 − JK705729. The six putative *S. chinensis* B-class genes were aligned with MADS-box genes from diverse angiosperm species [29] using Clustal-X [54]. Alignments were created from the relatively conserved MIK domain, while the C domain was excluded. A maximum likelihood (ML) tree was constructed based on the nucleotide alignments using the pairwise deletion option in MEGA5 [55]. Genetic distances were estimated under the Tamura-Nei model. Branch support was obtained through bootstrap analysis with 500 iterations.

### RNA *in-situ* hybridization

Developing inflorescences were fixed in FAA and dehydrated through a standard ethanol series. *In-situ* hybridization of *ScAP3-B* and *ScPI-B* transcripts was performed according to methods described previously [42]. Digoxigenin-labeled cDNA probes for *ScAP3-B* and for *ScPI-B* were derived from 94 bp- and 143 bp-long sequences, respectively, near the 3′ termini of the mRNAs including K3 helix and C-domain, and C-domain and 3′-UTR, respectively.

## Supporting Information

Figure S1 **Amino acid alignments of the K1 amphipathic α-helices of** ***APETALA3*** **homologues from** ***S. chinensis*** **and related species.** All loci are labeled on the right as to their genera or species of origin. Abbreviations: Art, *Aristolochia tomentosa*; Arm, *Ar. manshuriensis;* Are, *Ar. eriantha;* Arp, Ar. promissa; Tts, *Thottea siliquosa;* Ppn, *Piper nigrum;* Ph, *Peperomia hirta;* Aspl, *Asarum splendens;* Aspe, *Asarum speciosum;* Srh, *Saruma henryi;* Drw, *Drimys winterii;* Mp, *Magnolia praecocissima;* Ih, *Illicium henryi;* Nym, *Nymphaea sp.;* Am, *Amborella trichopoda,* Sc, *Saururus chinensis*.
(TIF)

Figure S2 **Amino acid alignments of the K2 and K3 amphipathic α-helices of** ***PISTILLATA*** **homologues from** ***S. chinensis*** **and related species.** Abbreviations: Art, *Aristolochia tomentosa*; Arm, *Ar. manshuriensis;* Are, *Ar. eriantha;* Arp, *Ar. promissa;* Tts, *Thottea siliquosa;* Ppn, *Piper nigrum;* Ph, *Peperomia hirta;* Aspl, *Asarum splendens;* Aspe, *Asarum speciosum;* Srh, *Saruma henryi;* Drw, *Drimys winterii;* Mp, *Magnolia praecocissima;* Ih, *Illicium henryi;* Nym, *Nymphaea sp.;* Am, *Amborella trichopoda,* Sc, *Saururus chinensis*.
(TIF)

Table S1 **qRT-PCR primers.**
(XLS)

Table S2 **Putative identifications of** ***S. chinensis*** **genes, and their transcript abundance levels in developing bracts, stamens, and carpels relative to their transcript abundance levels in developing seedling leaves.**
(XLS)
