# Supplementary figures and images for: Developmental Genetics of the Perianthless Flowers and Bracts of a Paleoherb Species, Saururus chinensis
Source: PLoS One. 2013 Jan 30;8(1):e53019. doi: 10.1371/journal.pone.0053019 (PMC3559744; doi:10.1371/journal.pone.0053019)

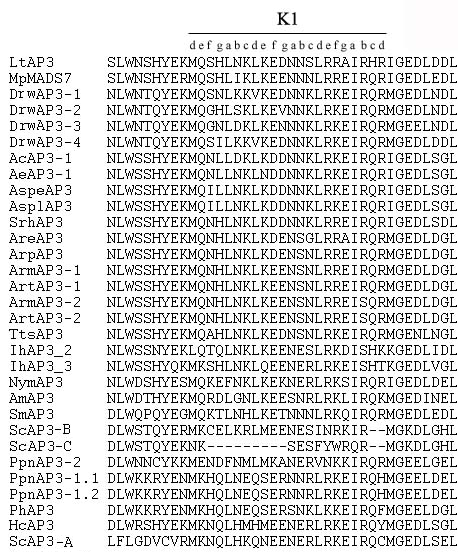

Supplement: Figure S1 — Amino acid alignments of the K1 amphipathic α-helices of APETALA3 homologues from S. chinensis and related species. All loci are labeled on the right as to their genera or species of origin. Abbreviations: Art, Aristolochia tomentosa; Arm, Ar. manshuriensis; Are, Ar. eriantha; Arp, Ar. promissa; Tts, Thottea siliquosa; Ppn, Piper nigrum; Ph, Peperomia hirta; Aspl, Asarum splendens; Aspe, Asarum speciosum; Srh, Saruma henryi; Drw, Drimys winterii; Mp, Magnolia praecocissima; Ih, Illicium henryi; Nym, Nymphaea sp.; Am, Amborella trichopoda, Sc, Saururus chinensis. (TIF) [file pone.0053019.s001.tif]

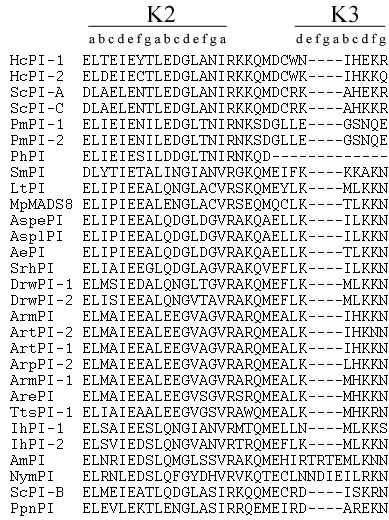

Supplement: Figure S2 — Amino acid alignments of the K2 and K3 amphipathic α-helices of PISTILLATA homologues from S. chinensis and related species. Abbreviations: Art, Aristolochia tomentosa; Arm, Ar. manshuriensis; Are, Ar. eriantha; Arp, Ar. promissa; Tts, Thottea siliquosa; Ppn, Piper nigrum; Ph, Peperomia hirta; Aspl, Asarum splendens; Aspe, Asarum speciosum; Srh, Saruma henryi; Drw, Drimys winterii; Mp, Magnolia praecocissima; Ih, Illicium henryi; Nym, Nymphaea sp.; Am, Amborella trichopoda, Sc, Saururus chinensis. (TIF) [file pone.0053019.s002.tif]
